# scCompressSA: dual-channel self-attention based deep autoencoder model for single-cell clustering by compressing gene–gene interactions

**DOI:** 10.1186/s12864-024-10286-2

**Published:** 2024-04-29

**Authors:** Wei Zhang, Ruochen Yu, Zeqi Xu, Junnan Li, Wenhao Gao, Mingfeng Jiang, Qi Dai

**Affiliations:** Zhejiang Sci-Tech University, Second Street 928, Hangzhou, Zhejiang 310018 China

**Keywords:** Single-cell RNA sequencing (scRNA-seq), Static gene–gene interactions, Coefficient compression, Dual-channel self-attention mechanism

## Abstract

**Background:**

Single-cell clustering has played an important role in exploring the molecular mechanisms about cell differentiation and human diseases. Due to highly-stochastic transcriptomics data, accurate detection of cell types is still challenged, especially for RNA-sequencing data from human beings. In this case, deep neural networks have been increasingly employed to mine cell type specific patterns and have outperformed statistic approaches in cell clustering.

**Results:**

Using cross-correlation to capture gene–gene interactions, this study proposes the scCompressSA method to integrate topological patterns from scRNA-seq data, with support of self-attention (SA) based coefficient compression (CC) block. This SA-based CC block is able to extract and employ static gene–gene interactions from scRNA-seq data. This proposed scCompressSA method has enhanced clustering accuracy in multiple benchmark scRNA-seq datasets by integrating topological and temporal features.

**Conclusion:**

Static gene–gene interactions have been extracted as temporal features to boost clustering performance in single-cell clustering For the scCompressSA method, dual-channel SA based CC block is able to integrate topological features and has exhibited extraordinary detection accuracy compared with previous clustering approaches that only employ temporal patterns.

**Supplementary Information:**

The online version contains supplementary material available at 10.1186/s12864-024-10286-2.

## Introduction

As a high-throughput technology, single-cell RNA sequencing (scRNA-seq) make it feasible to investigate the cellular heterogeneity and thus played a crucial role in systems biology and precision medicine. Distributions of cell subpopulations are closely related with cell states and disease subtypes. Cell clustering of scRNA-seq data is crucial to detect meaningful patterns from raw gene count matrix [1–3]. One important topic in single-cell data analysis is to decipher the cellular compositions and cell subpopulations of complex tissues [4, 5]. For instance, tumor-infiltrating immune cell compositions may play a role in understanding anti-tumor immune responses. Once the cell types were detected, temporal gene patterns were used to enhance the understandings of cell signatures. Compared with statistical approaches, deep learning models including graph learning and transformer have exhibited superior capability in analyzing high-dimensional single-cell transcriptomics data [6–8].

For single-cell transcriptomic data, feature selection was regarded as an essential step in extracting biologically meaningful patterns from raw count matrix [9, 10]. Without cell labels, unsupervised single-cell clustering methods were widely used to select informative genes, with the measure of highly variable genes (HVGs). Unsupervised M3Drop method selects genes whose dropout rate exceeds that of other genes as features [11]. Similarly, GiniCluster method employs a modified Gini index to detect genes whose expression is concentrated in a limited number of cells [12]. As an unsupervised method, the DUBStepR method defines a graph-based measure of cell aggregation in the feature space, and uses this measure to optimize the features [13]. Seurat method combines variance filtering and standardization to select genes with differential expression and large variance as inputs [14]. Meanwhile, the FEAST method selects genes with strong correlation to clustering results through mutual information filtering based on information entropy. In fact, topological features have played an increasingly important role in computational analysis of single-cell data [15, 16]. Gene–gene interactions can be regarded as topological features, thus capturing computational analysis of scRNA-seq data.

With extracted features, deep learning models including graph learning methods have been employed to extract meaningful patterns from raw gene count matrix [1, 17–19]. The scDeepCluster method combines depth-counting autoencoder (DCA) modeling and deep embedding to conduct single-cell clustering [20]. But high variable genes are not pre-selected in scDeepCluster, and lead to high time consumption and slowly increased clustering accuracy. In contrast, highly variable genes are selected as features in the scziDesk, thus reducing the computational burden as well as memory consumption. Both scDeepCluster and scziDesk employ a stack autoencoder (SAE) model to detect cell types and train a multi-layer autoencoder [21]. These deep learning methods use a stack autoencoder (SAE) which employs the CNN architecture. CNN model keeps the input neighborhood relationship and the spatial locality in the high-level feature representation, and effectively learn local features in input matrices. For single-cell data, CNN may encounter certain limitations. To alleviate these problems, deep convolutional autoencoder (CAE) has been used to replace SAE to learn the effective data compression.

Among various deep learning models, the autoencoder model has received increasing attention in the field of single-cell data analyzing. Autoencoder (AE) model refers to a type of neural network capable of effective data compression without supervision. This auto-encoder architecture conducts nonlinear dimension reduction of high-dimensional single-cell gene expression data in a latent space. The scCAEs method employs the convolutional autoencoder architecture and regularization terms designed for scRNA-seq data [22]. In the original scCAEs method, a multilayer convolutional autoencoder model is adopted to learn the low-dimensional representations of the input gene expression matrix. However, gene–gene interactions have not yet been taken into consideration during cell clustering. Actually gene–gene interactions can be regarded as topological features, thus contributing to a comprehensive model.

In order to enhance the performance of single-cell clustering, this study proposes a scCompressSA method to integrate expression patterns and gene–gene interactions, with self-attention (SA) based coefficient compression. The contributions of scCompressSA method are three-folds: F-test based selection of informative genes, coefficient compression (CC) based integration of gene–gene interactions, and dual-channel SA scheme based CC block. Two types of information, i.e. static gene–gene interactions and gene expression dynamics, are effectively integrated by the dual-channel SA based CC block, with the purpose of capturing spatial–temporal dynamics underlying RNA-sequencing data. Validation experiments about benchmark scRNA-Seq datasets are conducted to demonstrate the effectiveness and advantages of this scCompressSA method.

## Single-cell clustering using deep CAE architecture

In the conventional encoder-decoder (CAE) architecture, gene count matrices of individual cells are firstly reshaped into two-dimensional image that were employed to train deep neural networks. This reconstructed two-dimensional data matrix is able to learn non-linear gene–gene dependencies from complex and multi-cell type samples and guide the training of autoencoder model to construct embedded spaces that define cell types. In the deep CAE architecture, the expression profiles of individual cells are reshaped into two-dimensional (2D) data matrix and used as samples for model training.

### Data preprocessing of single-cell data

Assume $X$ as an unlabeled gene count matrix composed of $n$ samples, single-cell clustering approaches aim to divide these $n$ samples into $K$ categories. Gene count matrix was firstly transformed with a nonlinear mapping ${\phi }_{w}:X\to Z$, where $Z$ denotes a latent feature space with reduced dimension.

Given the top layer of the corrupted and clean pathway pathways as the embedding subspace, the polynomial logistic regression function is employed to predict the probability distribution. Soft label ${p}_{ik}$ of embedded point ${z}_{i}$ is defined by Eq. (1).

1 $${p}_{ik}=\frac{exp\left({\theta }_{k}^{T}{z}_{i}+{b}_{k}\right)}{\sum_{k=1}^{K}exp\left({\theta }_{k}^{T}{z}_{i}+{b}_{k}\right)}$$

where ${p}_{ik}$ represents the probability that the $i$-th cell is assigned to the $k$-th cluster, while ${{z}_{i}=\phi }_{w}\left({x}_{i}\right)\in Z$ represents the embedded ${x}_{i}\in Z$. For the $k$-th cell cluster, the set of weight vectors ${\theta }_{k}$ and bias values ${b}_{k}$ were computed. Deep clustering methods learned neural network classifier that maximizes the mutual information, which was converted to maximization of the clustering loss. Note that ${p}_{ik}$ is related with learnable parameters in neural network classifier. In soft clustering, the weight ${r}_{ik}$, which ranges from 0 to 1, denotes the weight of assigning the embedded data point ${z}_{i}$ to the $k$-th category.

2 $${r}_{ik}=\frac{exp\left(-\upbeta {\Vert {z}_{i}-{\mu }_{k}\Vert }^{2}\right)}{\sum_{k=1}^{K}exp\left(-\upbeta {\Vert {z}_{i}-{\mu }_{k}\Vert }^{2}\right)}$$

The total responsibilities of the respective point is 1, i.e. $\sum_{k=1}^{K}{r}_{ik}=1$. When the latent space $z$ and the responsibility ${r}_{ik}$ are known, the optima in a closed form can be obtained. Afterwards, the polynomial logistic regression function is used to predict the probability of cluster assignment ${p}_{ik}$.

### Training of deep CAE model

The original data $X$ is mapped to the embedded subspace $Z$, which contains $K$ clusters. In single-cell clustering, deep neural networks are trained with the loss function containing the K-means clustering target, which is defined by Eq. (3).

3 $${L}_{1}=\sum_{i=1}^{N}\sum_{k=1}^{K}{r}_{ik}{\left|\left|{z}_{i}-{\mu }_{k}\right|\right|}^{2}-\lambda \sum_{i=1}^{N}{z}_{i}^{T}{z}_{i}.$$

Furthermore, a reconstruction loss function is used as a data-dependent regularization during the training of deep neural networks, while a soft-max layer is superimposed on the CAE architecture to predict the soft allocation of clustering. In order to minimize the mismatch between the weight ${r}_{ik}$ and the probability distribution ${p}_{ik}$, the KL divergence is introduced into the objective function to reduce the distance between these two parameters.

4 $${L}_{2}=KL\left(R||P\right).$$

where $R$ and $P$ denote the set of target variables and predicted target probability ${p}_{ik}$ respectively. Here KL divergence plays the role of constraint to narrow the distance between predicted probability distribution and the soft distribution. In order to obtain a mapping function that is more suitable for K-means clustering, the squared error reconstruction loss ${L}_{3}$ between the decoder and encoder layers are introduced to the total loss function, which is defined by Eq. (5).

5 $${L}_{3}=\frac{1}{N}\sum_{i=1}^{N}\sum_{l=0}^{L-1}\frac{1}{\left|{z}_{i}^{l}\right|}{\left|\left|{z}_{i}^{l}-{\widehat{z}}_{i}^{l}\right|\right|}^{2}.$$

where $\left|{z}_{i}^{l}\right|$ denotes the output size of the $l$-th layer. The weigh ${r}_{ik}$ and the optima ${\mu }_{k}$ are alternately updated. Hence, the general loss function, consisting of three components, is optimized to learn network parameters.

6 $$L=\underset{W}{{\text{min}}}{L}_{1}+{\alpha }_{1}{L}_{2}+{\alpha }_{2}{L}_{3}.$$

where weighted coefficients ${\alpha }_{1}$ and ${\alpha }_{2}$ are employed to pursue a trade-off between two regularization terms. In this case, the reconstruction loss function of autoencoder model is employed as a data-dependent regularization term, with the purpose of avoid over-fitting.

### Evaluation metrics of cell clustering

For single cell clustering tasks, the performance is quantitatively evaluated by adjusted rand index (ARI) and normalized mutual information (NMI). Denote $U$ as the true partition of $P$ classes, while $V$ as the predicted partitions. In addition, ${n}_{i}$ and ${n}_{j}$ denote the number of the class ${\mu }_{i}$ and cluster ${v}_{j}$, respectively. ${n}_{ij}$ is represented as the number of observations in both class ${\mu }_{i}$ and cluster ${v}_{j}$. During evaluation, the ARI index is defined by Eq. (7).

7 $$ARI=\frac{\sum_{i=1}^{P}\sum_{j=1}^{K}\left(\genfrac{}{}{0pt}{}{{n}_{ij}}{2}\right)-\frac{\left[\sum_{i=1}^{P}\left(\genfrac{}{}{0pt}{}{{n}_{i\cdot }}{2}\right)\sum_{j=1}^{K}\left(\genfrac{}{}{0pt}{}{{n}_{\cdot j}}{2}\right)\right]}{\left(\genfrac{}{}{0pt}{}{n}{2}\right)}}{\frac{1}{2}\left[\sum_{i=1}^{P}\left(\genfrac{}{}{0pt}{}{{n}_{i\cdot }}{2}\right)\sum_{j=1}^{K}\left(\genfrac{}{}{0pt}{}{{n}_{\cdot j}}{2}\right)\right]-\frac{\left[\sum_{i=1}^{P}\left(\genfrac{}{}{0pt}{}{{n}_{i\cdot }}{2}\right)\sum_{j=1}^{K}\left(\genfrac{}{}{0pt}{}{{n}_{\cdot j}}{2}\right)\right]}{\left(\genfrac{}{}{0pt}{}{n}{2}\right)}}$$

where $n=\sum_{i=1}^{P}{n}_{i\cdot }=\sum_{j=1}^{K}{n}_{\cdot j}$. Meanwhile, the NMI index is expressed as Eq. (8).

8 $$NMI=\frac{2I\left(U,V\right)}{\left(H\left(U\right)+H\left(V\right)\right)}$$

where $I\left(U,V\right)$ is the amount of mutual information between $U$ and $V$, $H\left(U\right)$ and $H\left(V\right)$ are the entropy of partitions $U$ and $V$. In addition, clustering accuracy (ACC) is designed to measure the best matching between the predicted and true clusters, which is defined by Eq. (9).

8 $$ACC=\underset{m}{max}\sum_{i=1}^{n}1\frac{\left\{\widehat{{I}_{i}}=m\left({I}_{i}\right)\right\}}{n}$$

where $\widehat{{I}_{i}}$ and ${I}_{i}$ represent the true and predicted cell labels. It is noted that cell annotation and single-cell clustering tasks are similar while different in computational analysis of RNA-seq data. Single-cell clustering focus on difference in expression patterns of various cell types while cell annotation provide the specific functions of cell types.

## Self-attention based scCompressSA method

**Fig. 1:**
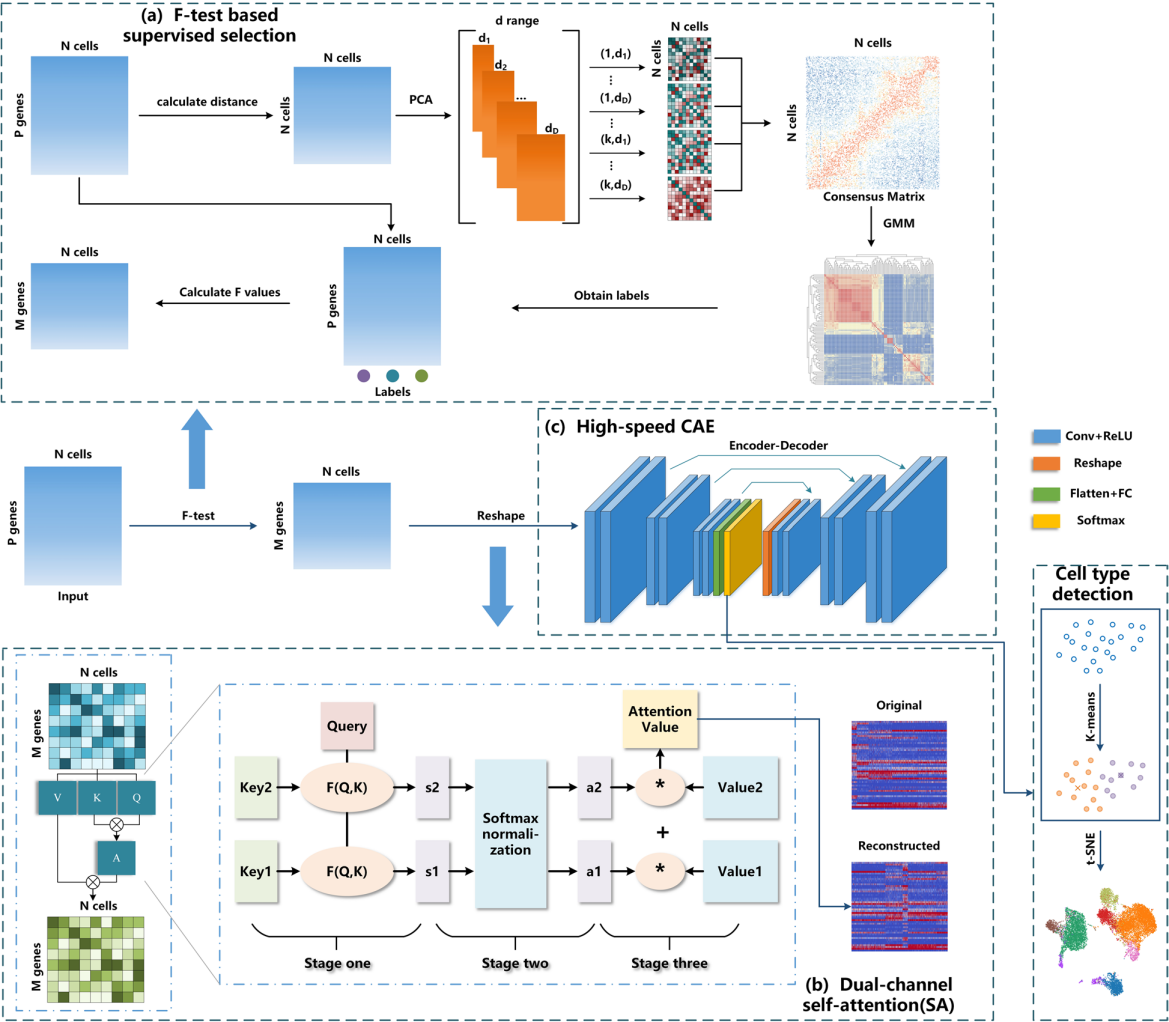
The diagram of self-attention based scCompressSA method. There are three blocks in the scCompressSA method, i.e. F-test based supervised learning, SA-based coefficient compression (CC) block and high-speed autoencoder architecture. The role of SA-based CC is to integrate static gene-gene interactions with temporal expression patterns

In order to detect cell types, this study proposes a self-attention mechanism based deep AE model to integrating expression pattern and gene–gene interactions. Cross-correlation values between transcript levels of gene pairs are computed to reconstruct input matrices. Reconstructed data matrix contains temporal expression patterns and gene–gene interactions. To assign the weighted coefficients, the scCompressSA employs automatic coefficient compression (CC) block quantitatively determine the contributions of these two parts in reconstructed input matrix. The architecture of scCompressSA method contains three blocks: F-test based supervised learning, SA-based CC block, an high-speed AE architecture. The architecture of scCompressSA method was demonstrated by Fig. 1.

In Fig. 1, three interconnected blocks have been designed to conduct single-cell clustering, with extracted topological features about gene-gene interactions. The role of section (a) is to perform F-test based supervised selection of informative genes, while the SA-based CC block in section (b) aims to integrate topological features with dual-channel self-attention mechanism. In this study, topological features, which correspond to static gene–gene interactions in gene expression matrix, were captured by cross-correlation between transcript levels and integrated by the SA mechanism into reshaped input matrix. The role of SA mechanism is to assign weights to two components of reconstructed input matrix according to their contributions.

### F-test based selection of informative genes

In selection of informative genes, the F-test method is used to compare significant differences among multiple cell samples. For each cell sample, *F*-value and *p*-value are calculated using analysis of variance (ANOVA). The ratio of within-group error to between-group error was used to evaluate whether there is a significant difference in means among the groups. In supervised selection of informative genes, F-test was used to conduct variance analysis, which is defined by Eq. (10).

10 $${F}_{\left(k-1,N-k\right)}=\frac{\frac{{\sigma }_{b}}{\left(k-1\right)}}{\frac{{\sigma }_{w}}{\left(N-k\right)}}$$

In Eq. (10), ${F}_{\left(k-1,N-k\right)}$ represents the degrees of freedom in $F$ distribution, where $k$ denotes the number of groups and $N$ is the total sample size. After computing the *F*-value, *p*-value for each feature is computed using the $F$ distribution with degrees of freedom of $\left(k-1,N-k\right)$. The formula for calculating the *p*-value is defined by Eq. (11).

11 $$p=1-{F}_{\left(k-1,N-k\right)}\left(F\right)$$

F-test, which was employed in informative gene selecting, is associated with correlation level between each feature and its corresponding category by comparing the ratio of variances. By analyzing the contribution of mark genes to the response variable variance, F-test identify the most informative genes for cell clustering, which is described by Eq. (12).

12 $${F}_{i}=\frac{{\sigma }_{b}}{{\sigma }_{w}}$$

The variable ${\sigma }_{b}$ represents the variance between different groups, while ${\sigma }_{w}$ denotes the variance within each group. In the application of gene expression matrices, ${\sigma }_{b}$ was regarded as the differences in gene expression between different categories, while ${\sigma }_{w}$ is related with the degree of fluctuation in gene expression.

### Self-attention based coefficient compression (CC)

The scCompressSA method designs and implements self-attention based coefficient compression (CC) to integrate static gene–gene interactions with temporal expression patterns. Correlation values of transcript levels between gene pairs were calculated to capture the dynamics of gene–gene interactions. Considering the characters of RNA-seq data, dual-channel self-attention mechanism was employed to assign suitable weights for topological and temporal patterns in reconstructed input matrix.

The two-channel SA mechanism embedded in coefficient compression is illustrated in the Fig. 1(b). In this dual-channel SA architecture, In this dual-channel SA, there are three inter-connected stages. The first stage aims to calculate the cross-correlation ${s}_{i}$ between *Query* and Key value${K}_{i}$, while the second stage computes the coefficients ${a}_{i}$ of ${K}_{i}$ by standardizing ${s}_{i}$ through softmax function. Eventually, the third stage in dual-channel SA scheme computes attention scores to determine weights for temporal and topological features in reconstructed data matrix.

In this case, topological features underlying transcriptomic data take the form of cross-correlation between transcript levels of gene pairs. Cross-correlation values between gene-pairs is defined by Eq. (13).

13 $${c}_{k}=\sum_{n}\left({a}_{n+k}\cdot {\overline{v}}_{n}\right)$$

In Eq. (13), the sequence $a$ is first unified to the length of n + k, and if the length is not enough, zero padding is performed. ${\overline{v}}_{n}$ is the complex conjugate of ${v}_{n}$. In this self-attention (SA) architecture, $a$ *and *$v$* represent the same sequence, namely *$x$*. Therefore, the autocorrelation of *$k$*-th gene in a cell are calculated according to Eq. *(14).

14 $${x}_{k}=\sum_{n}\left({x}_{n+k}\cdot {\overline{x}}_{n}\right)$$

Denote $x$ as a cell, then ${x}_{i}$ represents the expression of the $i$-th gene in the cell. Decompose the gene expression ${x}_{i}$ into two components which are assigned with coefficients $\alpha$ and $\beta$ respectively, for specific decomposition, see Eq. (15). IIn reconstructed gene expression matrix $X$, where ${X}_{ij}\left(1\le i\le n,1\le j\le p\right)$ indicates the expression of $j$-th gene in the $i$-th cell of $X$.

In this case, reconstructed data matrices contain temporal expression dynamics and topological features, which correspond to nonlinear gene–gene interactions. The specific formula of reconstructed input matrix is computed according to Eq. (15).

15 $${x}_{i}=\alpha {x}_{i}+\beta \frac{\sum_{j=1}^{n}\left({x}_{j}\cdot {x}_{j+i}\right)}{n},i=\mathrm{1,2},\dots ,n.$$

In Eq. (15), the second part $\frac{\sum_{j=1}^{n}\left({x}_{j}\cdot {x}_{j+i}\right)}{n}$ refers to the cross-correlation between gene pairs. In this case, static gene–gene interactions were extracted and incorporated into the reconstructed input matrix. Given a specific scRNA-seq dataset, the attention mechanism has been employed to find optimal or suboptimal combination of gene expression and gene–gene interaction components.

The attention function was described as mapping a query and a set of key-value pairs to an output. Transcript levels of informative gene ${x}_{i}$ in each cell are regarded as *Query* values, and two parts ${x}_{i}$ and $\frac{\sum_{j=1}^{n}\left({x}_{j}\cdot {x}_{j+i}\right)}{n}$ are regarded as ${K}_{1}$ and ${K}_{2}$. In subsequent computation, attention matrix $F\left(Q,{K}_{i}\right)$ is computed by $Q$ and the corresponding $K$, scaled by the inverse of the square of dimension of $K$ (${d}_{k}$) and activated by softmax function.

16 $$F\left(Q,{K}_{i}\right)=softmax\left(\frac{{K}_{i}^{T}Q}{\sqrt{{d}_{k}}}\right)$$

where ${d}_{k}$ denotes the dimension of input vectors. The similarity values of *Query* and *Key* varies depending on the selected computational mechanism. A modified softmax mechanism has been employed to to covert similarity value and organize the scores into probability distributions. The formula for normalizing similarity values is defined by Eq. (17).

17 $${a}_{i}=Softmax\left(F\left(Q,{K}_{i}\right)\right)=\frac{{e}^{F\left(Q,{K}_{i}\right)}}{\sum_{j=1}^{{L}_{x}}{e}^{F\left(Q,{K}_{j}\right)}}$$

In Eq. (17), ${a}_{i}$ denotes the weighted coefficient corresponding to attention scores. In the scCompressSA method, correlation values of between gene pairs are computed to capture static gene–gene interactions. The final formula to calculate the weights is defined as follows:

18 $$\begin{array}{c}\alpha =Softmax\left(F\left({x}_{i},{x}_{i}\right)\right)\\ =\frac{exp\left(F\left({x}_{i},{x}_{i}\right)\right)}{exp\left(F\left(\frac{{\sum }_{j=1}^{n}\left|{x}_{i}\mp {x}_{j}\right|}{n},{x}_{i}\right)\right)+exp\left(F\left({x}_{i},{x}_{i}\right)\right)}\end{array}$$

19 $$\begin{array}{c}\beta =Softmax\left(F\left(\frac{{\sum }_{j=1}^{n}\left|{x}_{i}-{x}_{j}\right|}{n},{x}_{i}\right)\right)\\ =\frac{exp\left(F\left(\frac{{\sum }_{j=1}^{n}\left|{x}_{i}-{x}_{j}\right|}{n},{x}_{i}\right)\right)}{exp\left(F\left(\frac{{\sum }_{j=1}^{n}\left|{x}_{i}-{x}_{j}\right|}{n},{x}_{i}\right)\right)+exp\left(F\left({x}_{i},{x}_{i}\right)\right)}\end{array}$$

Each cell recalculates the coefficients to reconstruct the matrix. Such computation has the advantage of greatly saving the time cost of manual parameter reconstruction matrix and achieving an optimal result. With the weighted coefficients of *Key* values, the attention scores in SA scheme are computed by the sum operation.

20 $$A=\sum_{i=1}^{{L}_{x}}{a}_{i}\cdot {V}_{i}$$

Reconstructed data matrix *A* consists of two components, i.e. gene expression and static gene–gene interaction dynamics. The architecture of deep autoencoder model has been optimized to reduce computational burden without significant loss of clustering accuracy.

In the scCompressSA method, SA-based CC block aims to integrate static gene–gene interactions into the reconstructed input matrix. The function of $s$ balances the contributions of temporal expression dynamics and nonlinear gene–gene interactions to compressed data matrix. The value of probability density ${p}_{ik}$ denotes the probability that $i$-th cell is assigned to the $k$-th cell cluster.

## Experimental outcomes and analysis

In the validation experiment, multiple scRNA-Seq datasets with cell annotations have been selected as benchmarks to evaluate the performance of scCompressSA and candidate cell clustering methods. These scRNA-Seq data were downloaded from multiple sequencing platforms including 10X genomics and GEO, which contain expression profiling by high throughout sequencing technology.

Nine benchmark scRNA-seq datasets with cell type labels are used to validate the performance of clustering approaches. Among these datasets, five groups of peripheral blood mononuclear cells (PBMCs) datasets have been selected as benchmarks in the clustering experiments. These PBMC datasets were measured from multiple platforms. Details of these scRNA-seq datasets are given in Table 1.

**Table 1:** Descriptions of benchmark scRNA-seq datasets with cell labels

Datasets	Clusters	Cells	Genes	Sample size	Platform
Zeisel	9	3005	19972	115MB	Illumina
Klein	4	2717	24047	249MB	inDrop
Petropoulos	4	1529	21749	82.1MB	Drop-seq
AD-brain	8	13214	10852	273MB	Illumina
PBMC-Kang-A	8	11432	14504	316MB	10X
PBMC-Kang-B	8	12261	14473	339MB	10X
PBMC-Kang-C	8	11989	14222	325MB	10X
PBMC-Ding	10	7111	20428	557MB	10X
PBMC-Zheng4k	8	4340	33694	279MB	10X

In Table 1, 10X denotes the platform of 10X Chromium. Among these scRNA-seq datasets with cell labels, PBMC-Zheng4k denotes the blood expression data from human. For the PBMC-Kang-A data, HiSeq 2500 data was used for sequencing of PBMC-Kang-A from SLE patients and 2 controls. 1 M cells were collected from frozen PBMC-Kang-A samples that were prepared using the 10 × single cell instrument according to standard protocol. PBMC -Kang-A, B, and C were prepared on the instrument directly following thaw.

### F-test based supervised selection of informative genes

**Fig. 2:**
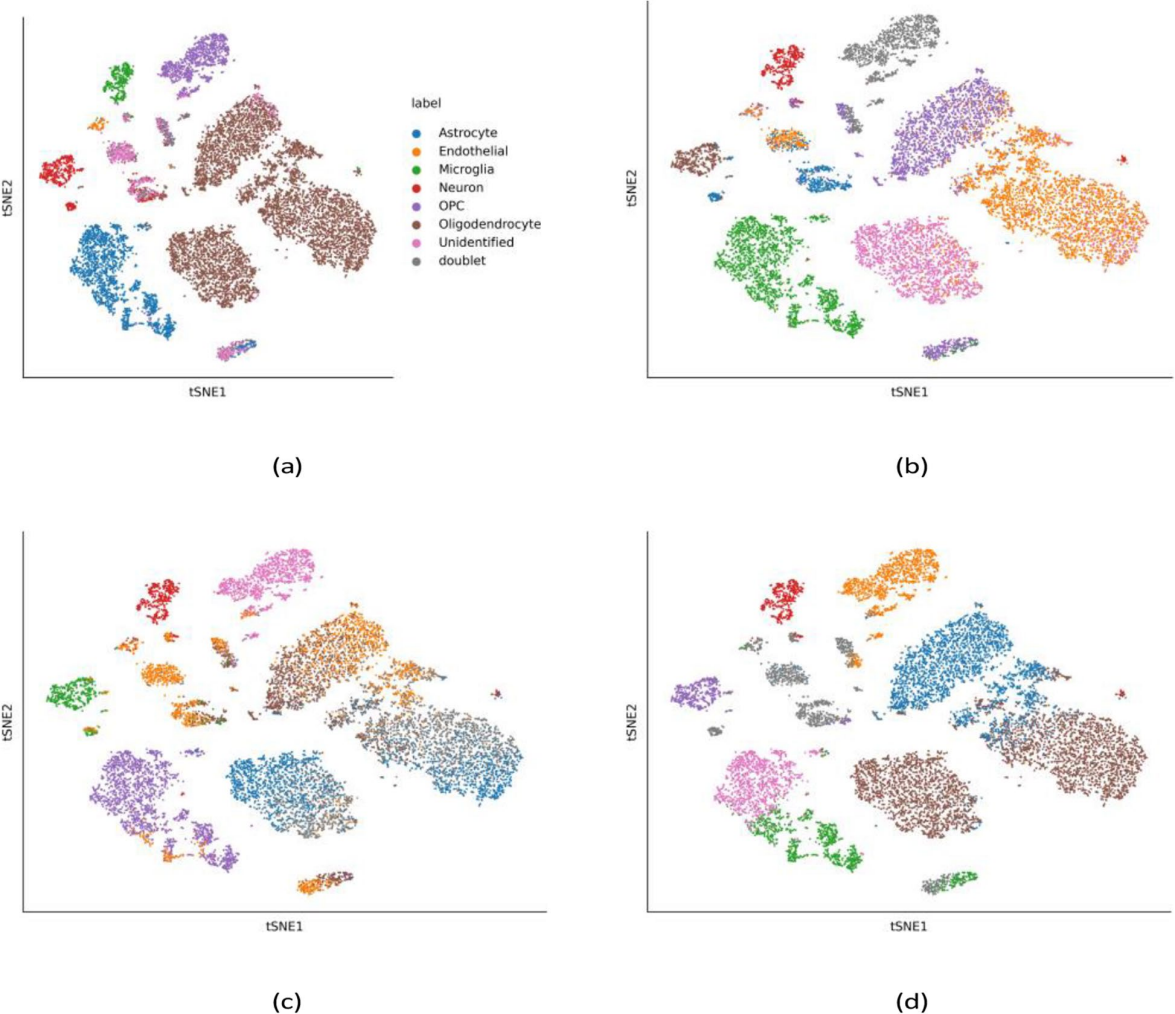
Distributions of cell subclusters and ground truths of AD-brain data in the feature space with reduced dimension. Unsupervised and supervised learning were compared in cell clustering. **a** Ground truths of cell types in the AD-brain data; **b** Unsupervised clustering predicted distributions of cell sub-clusters; **c** HVG-based supervised selection; **d** F-test based supervised selection

In F-test based supervised learning, the scCompressSA approach employs the SelectKBest function to select top *k* features based on the *F*-value computed from single cell expression data. The AD-associated brain expression data, which was denoted as AD-brain, was collected from human brains of 12 individuals, yielding 13,214 high quality nuclei. For the AD-brain data, predicted distributions of cell types and the ground truths are visualized and compared by Fig. 2.

For AD-brain data, subpopulations of astrocyte and oligodendrocyte progenitor cells were hypothesized to play a crucial role in regulating disease progression. Shown in Fig. 2, F-test based supervised selection outperforms HVG-based selection. Compared with unsupervised learning, cell types detected by supervised clustering method show higher consistency with ground truths.

**Fig. 3:**
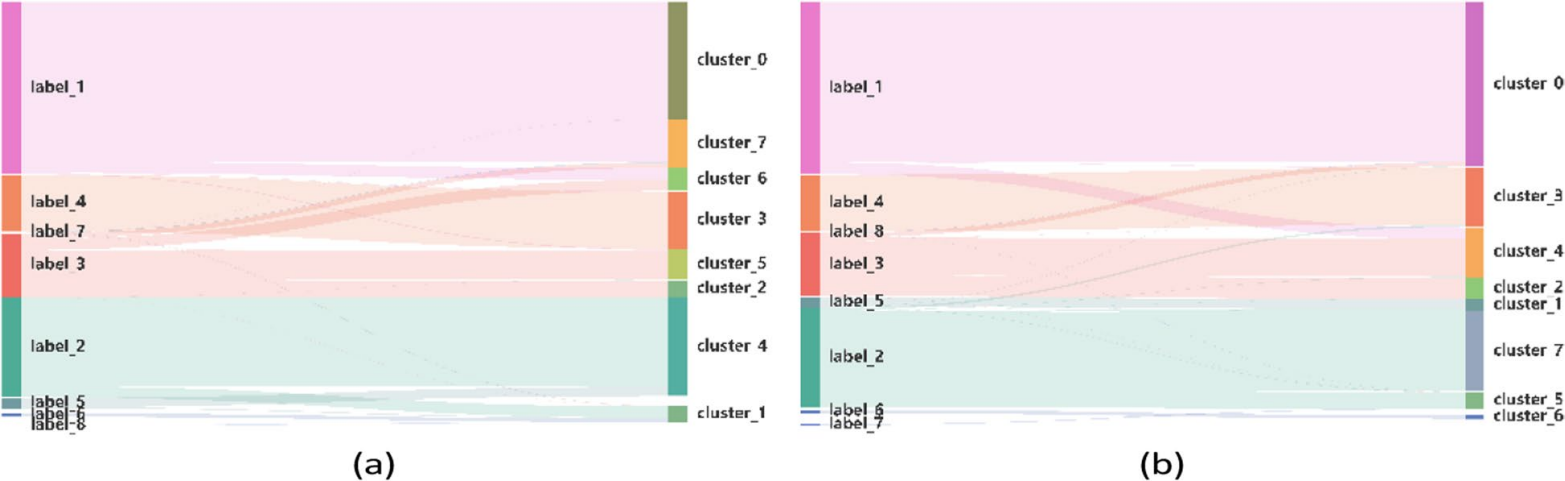
Sankey plots of cell clustering outcomes for the PBMC-Zheng4K dataset. **a** Cell types predicted by HVG-based gene selection; **b** Predictions of cell types by F-test based supervised learning

Meanwhile, the accuracy metrics of multiple cell clustering approaches were demonstrated by Sankey plot. Using HVG and F-test based feature selection, Sankey plots of cell clustering for PBMC-Zheng4k data are compared in Fig. 3.

For PBMC-Zheng4k data, clustering metrics obtained by F-test based supervised learning are computed as (ari = 0.824, nmi = 0.791, acc = 0.862) respectively, while the metrics obtained by HVG-based feature selection methods are (ari = 0.519, nmi = 0.645, acc = 0.669), using the same autoencoder framework. This improvement indicates that F-test supervised learning exhibited enhanced performance than conventional HVG-based method.

### Integration of static gene–gene interactions

In the scCompressSA method, the coefficient compression (CC) block integrates gene-gene interactions and gene expression patterns to reconstruct input matrices. This CC block aims to capture static gene–gene interactions by computing correlation values of transcript levels between gene pairs. In this way, reconstructed input matrices contain two components of dynamic information and will be fed into the deep neural network models to detect cell type-specific patterns. Two types of compression methods, namely fixed-parameter CC, and self-attention based CC, were considered. This section firstly investigates the fixed-parameter coefficient compression.

To explore the role of coefficients, the experiment conducted multiple groups of cell clustering for the PBMC-Zheng4k dataset using various fixed *α* values. Relevant results are depicted in the diagram below. Herein, *α* = 1 corresponds to single data modality of gene expression.

**Fig. 4:**
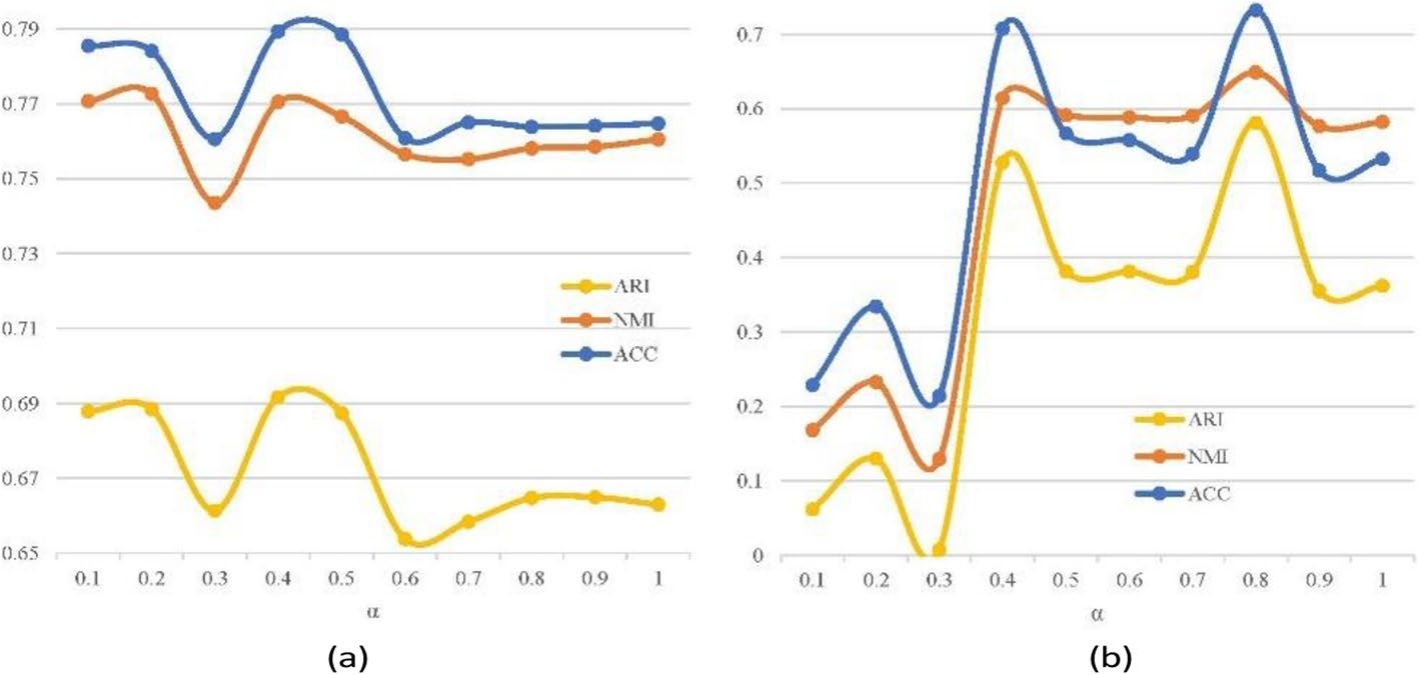
Impact of weight selection in data compression on clustering metrics for PBMC-Zheng4k and AD-brain datasets. **a** denotes clustering outcomes of PBMC-Zheng 4 k data, **b** corresponds to AD-associated brain expression data. Selection of weighted coefficients *α* has played an essential role in integrating two components in reconstructed input matrices

In Fig. 4, significant disparity have been observed in the accuracy metrics obtained using different coefficients. For two groups of scRNA-seq datasets, there existed optimal or sub-optimal combination of weights to balance gene expression dynamics and gene–gene interactions. In order to find the optimal combination of weights, the proposed scCompressSA method adopts self-attention (SA) mechanism in coefficient compression to integrate two types of dynamics, i.e. gene expression dynamics and gene–gene interactions. This dual-channel SA mechanism automatically assigns weights and obtains the reconstructed input to train deep autoencoder model.

Evaluation metrics obtained under two situations have demonstrated the effectiveness and advantages of SA-based coefficient compression. Cell clustering using single modeling perspective of RNA-seq data can capture local information about cell types.

Single-cell clustering performance has been enhanced by integrating static gene–gene interactions with coefficient compression.

### Dual-channel self-attention based compression strategy

Although the coefficient compression (CC) block improves cell type detection accuracy to some extent, the process rely heavily on manually specified coefficients, leading to computational inconvenience and unstable outcomes. In this sector, dual-channel self-attention (SA) mechanism is adopted automatically allocates coefficients to two components, thus reconstructing input matrices for deep CAE networks. During the coefficient compression, the dual-channel SA mechanism learns static functional interactions between different genes. It automatically assigns suitable weights according to transcript levels of gene pairs, with the purpose of capturing spatial information underlying RNA-sequencing data. By multiplying the weights learned from the attention layer with count matrix of informative genes, the final reconstruction matrix is obtained.

This SA-based CC block reconstructs input matrix by integrating static gene–gene interactions and subsequently fed into deep CAE model. Visualization of cell sub-populations demonstrates that predicted cell subpopulations that are highly consistent with ground truths. The ensuing diagram compares the performance of predicted cell types with the clustering outcomes obtained by temporal perspective only. Two groups of RNA-seq datasets, which were measured from human brain samples, have been employed to demonstrate the effectiveness and advantages of SA-based CC strategy.

In Table 2, 'Fixed CC' denotes fixed-parameter compression strategy. The weighted coefficient $\alpha$ was automatically allocated to balance the contributions of temporal and topological perspectives, i.e. gene expression dynamics and gene–gene interactions. Under this circumstance, the dual-channel SA mechanism has played the role of searching the optimal balance point between two modeling perspectives. This SA-based CC block embedded in the scCompressSA method is able to automatically assign weights based on interactions between gene pairs, thus integrating topological features in single-cell data.

**Table 2:** Ablation study of SA-based compression strategies on two groups of AD-associated single-cell expression data

Datasets	Strategy	NMI	ARI	ACC
NC-brain	Fixed CC	0.756	0.591	0.677
	SA-based CC	**0.800**	**0.650**	**0.734**
AD-brain	Fixed CC	0.488	0.348	0.487
	SA-based CC	**0.542**	**0.413**	**0.515**

**Fig. 5:**
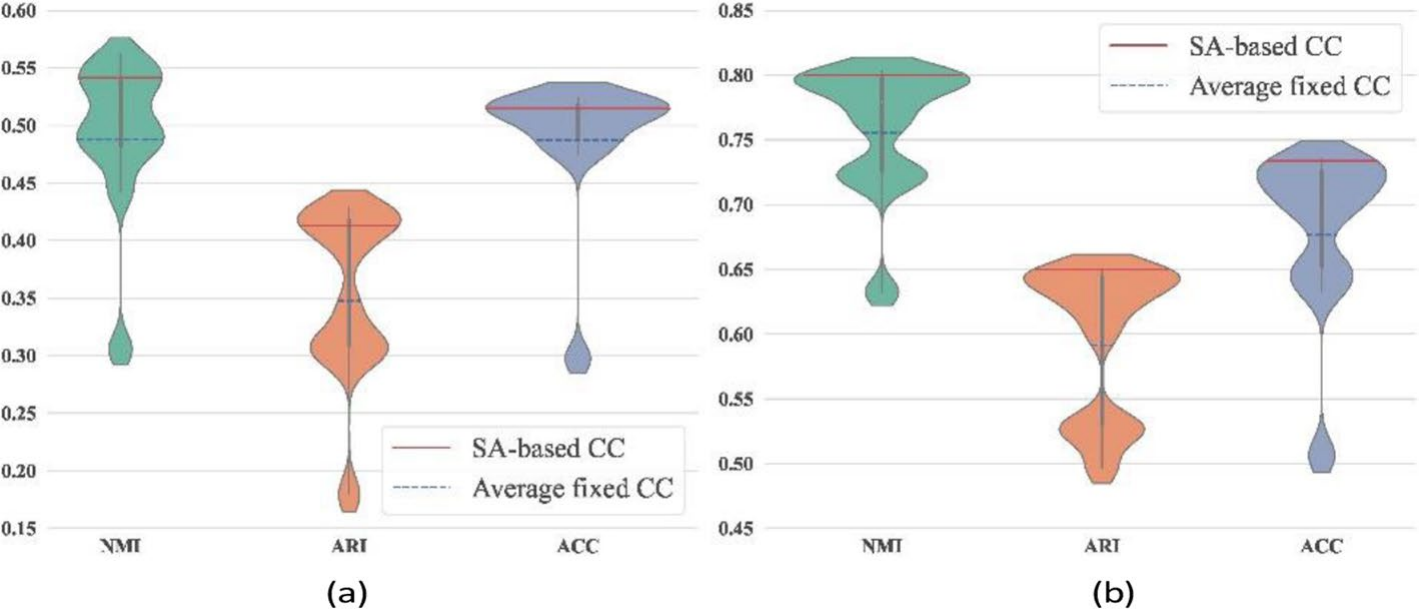
Comparison of two compression strategies for AD-associated brain expression data. Experimental outcomes were obtained through 12 replicate experiments on two groups of scRNA-seq datasets: **a** Healthy group expression data (HC-brain); **b** AD associated brain expression data (AD-brain). In violin plot, the label’fixed CC’ denotes the average indexes obtained by ten replicated experimental outcomes of fixed-parameter compression strategy, while SA-based CC represents the clustering indexes of dual-channel SA-based compression strategy

To further investigate the characteristics of dual-channel SA, SA-based CC and fixed-compression strategies (fixed CC) were implemented and compared. In ablation experiments, violin plots are used to illustrate the effectiveness of SA-based CC strategy, shown in Fig. 5.

From Fig. 5, it can be found that the SA-based CC strategy exhibits enhanced accuracy than fixed CC strategy in single-cell clustering tasks. The blue dashed line in violin plots represents the average of cell type detection using fixed CC strategy, while the red dashed line represents predictions obtained by SA-based CC. Although the SA mechanism yields sub-optimal solutions in specific cases, it still outperforms fixed CC strategy. In addition, fixed CC requires manually specifying coefficients, which is expected to consume considerable time costs.

### Performance evaluation of cell clustering methods

In order to quantitatively evaluate the performance of cell clustering method, benchmark scRNA-seq datasets with cell labels have been employed in single-cell clustering experiments. In this study, total ten groups of labeled scRNA-Seq datasets including five PBMCs have been used as benchmarks. Multiple deep learning based clustering approaches include Seurat, SC3, scCAEs methods are used as SOTA methods. Evaluation metrics of the scCompressSA method and other SOTA algorithms are calculated and compared in Table 3.

**Table 3:** Performance comparison of the scCompressSA method and other SOTA clustering approaches. Average clustering metrics and deviation values in replicate experiments have been recorded for multiple scRNA-seq datasets with cell type labels

Datasets	Metrics	Seurat	SC3	scCAEs	scCompressSA
Zeisel	ARI	0.507 (± 0.071)	0.628 (± 0.198)	0.640 (± 0.075)	0.8**03 (± 0.025)**
	NMI	0.666 (± 0.019)	0.704 (± 0.061)	0.668 (± 0.045)	0.7**66 (± 0.016)**
	ACC	0.665 (± 0.054)	0.707 (± 0.145)	0.778 (± 0.054)	0.8**80 (± 0.020)**
Klein	ARI	0.528 (± 0.024)	0.586 (± 0.004)	0.725 (± 0.048)	0.7**32 (± 0.057)**
	NMI	0.743 (± 0.017)	0.774 (± 0.012)	0.731 (± 0.028)	0.7**45 (± 0.032)**
	ACC	0.574 (± 0.015)	0.641 (± 0.010)	0.746 (± 0.074)	0.7**74 (± 0.099)**
Petropoulos	ARI	0.332 (± 0.001)	0.363 (± 0.090)	0.434 (± 0.029)	0.4**63 (± 0.002)**
	NMI	0.554 (± 0.011)	0.572 (± 0.010)	0.378 (± 0.124)	0.5**73 (± 0.011)**
	ACC	0.482 (± 0.001)	0.458 (± 0.025)	0.607 (± 0.020)	0.7**12 (± 0.044)**
NC-brain	ARI	0.570 (± 0.004)	0.358 (± 0.011)	0.634 (± 0.019)	0.8**35 (± 0.015)**
	NMI	0.787 (± 0.003)	0.423 (± 0.002)	0.774 (± 0.015)	0.8**34 (± 0.016)**
	ACC	0.651 (± 0.008)	0.212 (± 0.016)	0.728 (± 0.028)	0.7**81 (± 0.031)**
AD-brain	ARI	0.270 (± 0.016)	0.352(± 0.039)	**0.356 (± 0.034)**	0.343 (± 0.054)
	NMI	0.517 (± 0.004)	0.228 (± 0.005)	0.516 (± 0.011)	0.5**23 (± 0.025)**
	ACC	0.433 (± 0.006)	0.238 (± 0.012)	0.483 (± 0.020)	0.5**02 (± 0.013)**
PBMC-Kang-A	ARI	0.571 (± 0.056)	0.323 (± 0.025)	0.661 (± 0.079)	0.7**49 (± 0.107)**
	NMI	0.728 (± 0.019)	0.273 (± 0.063)	0.707 (± 0.035)	0.7**34 (± 0.019)**
	ACC	0.701 (± 0.043)	0.359 (± 0.058)	0.755 (± 0.046)	0.7**96 (± 0.042)**
PBMC-Kang-B	ARI	0.527 (± 0.045)	0.564 (± 0.009)	0.660 (± 0.050)	0.6**96 (± 0.026)**
	NMI	0.694 (± 0.016)	0.458 (± 0.004)	0.671 (± 0.046)	0.7**07 (± 0.019)**
	ACC	0.641 (± 0.049)	0.309 (± 0.007)	0.7**06 (± 0.038)**	0.701 (± 0.015)
PBMC-Kang-C	ARI	0.524 (± 0.003)	0.325 (± 0.010)	0.589 (± 0.008)	0.7**01 (± 0.028)**
	NMI	0.693 (± 0.002)	0.282 (± 0.020)	0.700 (± 0.017)	0.7**14 (± 0.007)**
	ACC	0.667 (± 0.032)	0.373 (± 0.034)	0.696 (± 0.016)	0.7**25 (± 0.009)**
PBMC-Ding	ARI	0.390 (± 0.009)	0.282 (± 0.070)	0.416 (± 0.026)	0.4**53 (± 0.015)**
	NMI	0.6**08 (± 0.004)**	0.459 (± 0.056)	0.546 (± 0.005)	0.566 (± 0.008)
	ACC	0.532 (± 0.001)	0.455 (± 0.022)	0.556 (± 0.055)	0.6**28 (± 0.020)**
PBMC-Zheng4k	ARI	0.629 (± 0.003)	0.577 (± 0.103)	0.663 (± 0.019)	0.6**64 (± 0.007)**
	NMI	0.756 (± 0.004)	0.706 (± 0.049)	0.761 (± 0.016)	0.7**63 (± 0.007)**
	ACC	0.718 (± 0.002)	0.649 (± 0.091)	0.7**50 (± 0.013)**	0.749 (± 0.013)

For multiple groups of brain and blood expression datasets, the proposed scCompressSA method has dramatically improved clustering accuracy than previous deep learning models. For two groups of brain expression data, the scCompressSA method has obtained superior clustering performance over existing approaches. This phenomenon indicates that gene–gene interactions was valuable to explore distributions of neuronal cell types that are associated with Alzheimer’s disease. Evaluation metrics of ARI, NMI and ACC demonstrated that the scCompressSA method outperforms cutting-edge algorithms such as scCAEs and SC3, in multiple datasets including brain expression and PBMCs datasets. Such enhanced capability of the scCompressSA method are crucial to conduct molecular diagnosis as well as disease progression modeling using single-cell transcriptomics profiles.

According to Table 3, topological features have played a unique role in downstream analysis of RNA-seq data. Such spatial dynamics could be integrated by the dual-channel SA mechanism, which assigns weights to two components of reconstructed data matrix with regards to their contributions. Compared with deep CAE method, this scCompressSA method is highly computationally efficient by implementing high-speed CAE network architecture. It seems that the scCompressSA method has achieved a balance between accuracy and efficiency in single-cell clustering tasks.

## Discussion

F-test based supervised selection aims to select informative genes for downstream analysis of RNA-seq data. This supervised selection block is believed to provide high-quality data matrices for subsequent model training by discarding low-quality cells. Reads that are obtained from the remaining cells are then normalized to compute the distance between cell pairs in feature space. During the training of scCompressSA, deep autoencoder architecture has been employed to detect cell types by integrating temporal as well as topological features. In this work, topological features correspond to functional interactions between genes.

However, there are also some inherent limitations to the original deep CAE model. This CAE architecture may not perform well on data with complex global structures, as it tends to focus on local features. In addition, the biological meanings of learned representations are still unclear, as the filters in the convolutional layers may not correspond directly to meaningful features in the input data. According to experimental outcomes, the performance of deep CAE model seem depend heavily on the choice of hyper parameters, including the number of layers and the size of the filters, which are difficult to optimize.

According to experimental outcomes, the scCompressSA method is able to alleviate these limitations encountered by conventional deep CAE models by introducing gene-gene interaction information with SA mechanism. The contribution of the proposed scCompressSA method is three-folds: supervised selection of informative gene sets, integration of gene–gene interactions, dual-channel SA-based CC. Dual-channel SA scheme was designed with regard to the characteristics of RNA sequencing data and has exhibited high computational efficiency. In addition, this dual-channel SA-based CC effectively boosts detection performance in single-cell clustering than the fixed-parameter CC block.

### Supplementary Information

**Additional file 1.** Evaluation metrics of replication experiments obtained by the scCompressSA and SOTA single-cell clustering approaches, including Seurat, SC3, scCAEs. Ten groups of benchmark scRNA-seq datasets with cell type labels have been employed in model comparison. Among these datasets, multiple PBMCs data from human were investigated in this study.

## Data Availability

Zeisel dataset was collected from hippocampus of mouse brain tissue with 3005 cells (Access number: GSE60361). Chen data was obtained from single-cell RNA sequencing of adult mouse hypothalamus using the Drop-seq platform (Access number: GSE87544). Klein dataset was measured from mouse embryonic stem cells using inDrop technology (Access number: GSE65525). This Klein case contains 2717 cells that are divided into 4 cell types. The Petropoulos dataset (Access number: E-MTAB-3929) was an scRNA sequencing dataset with time series (https://www.ebi.ac.uk/biostudies/arrayexpress/studies/E-MTAB-3929). Multiple groups of blood expression datasets were used in clustering experiments. PBMC-Kang-A, PBMC-Kang-B, PBMC-C were sequenced from peripheral blood of healthy individuals by Kang (Access number: GSE96583), while PBMC-Ding was sequenced from peripheral blood of individuals with disease (https://singlecell.broadinstitute.org/single_cell/study/SCP424/single-cell-comparison-pbmc-data). PBMC-Zheng4K, which was downloaded from 10X Genomics platform (https://support.10xgenomics.com/single-cell-gene-expression/datasets/2.1.0/pbmc4k), are expression data of peripheral blood mononuclear cells from healthy donors . For these scRNA-seq datasets, pre-processed count matrix and cell type labels are available and have been uploaded to the Zenodo platform. Benchmark scRNA-seq datasets could be downloaded from https://zenodo.org/record/8256590.
